# Post‐transcriptional regulatory networks: The dynamic interplay of RNA‐binding proteins

**DOI:** 10.1111/febs.70524

**Published:** 2026-04-01

**Authors:** Lena A. Street, Marko Jovanovic, Eugenio F. Fornasiero

**Affiliations:** ^1^ Department of Biological Sciences Columbia University New York NY USA; ^2^ Institute of Neuro‐ and Sensory Physiology University Medical Center Göttingen Germany; ^3^ Department of Life Sciences University of Trieste Italy

**Keywords:** post‐transcriptional regulation, RNA‐binding proteins, RNA–protein interactions, RNP complex dynamics, systems biology of RNA regulation

## Abstract

Post‐transcriptional regulation of gene expression is orchestrated by RNA‐binding proteins (RBPs), which regulate key aspects of the RNA life cycle including splicing, localization, translation, and decay. Although RBPs have been initially considered as isolated regulators, it is becoming clear that RNA molecules are commonly bound by several RBPs whose coordination directs their fate. These combinatorial interactions produce complex, context‐dependent post‐transcriptional regulatory networks (PTRNs) whose outcomes are difficult to predict. RBPs may also switch function depending on cell state, subcellular localization, or post‐translational modification, adding further complexity to RNA regulation. This review focuses on recent technological advances expanding our ability to map and interpret PTRNs. Multiplexed methods allow profiling of the RNA‐binding patterns of several RBPs in parallel, whereas deeper interaction proteomics studies reveal protein–protein connections and changes in distinct biological settings. Complementary RNA‐targeting pulldown and single‐molecule imaging strategies enable real‐time and single‐cell‐resolution visualization of ribonucleoprotein assembly and dynamics, while functional high‐throughput screens allow assignment of first order functions for these RBPs. Overall, these approaches set the stage for comprehensive decoding of the spatiotemporal structure of PTRNs and reveal how RBP interactions coordinate sets of RNAs to collectively regulate them in response to physiological demands. In addition to describing these systems‐level approaches, we outline key future analytical and experimental innovations that could transform our understanding of RBP function. We believe that a systems‐level understanding of RBPs as dynamic, integrated components of multiscale regulatory regimes is required to fully understand the complexity of gene expression control and its disruption in disease.

## Graphical abstract

Post‐transcriptional gene expression regulation is controlled by RNA‐binding proteins (RBPs), which influence many processes including splicing, localization, translation, and decay. Rather than acting alone, RBPs bind RNAs combinatorially, forming complex, context‐dependent regulatory networks. Recent advances, including multiplexed RBP–RNA‐binding assays, interaction proteomics, RNA pulldowns, imaging, and high‐throughput screens, are enabling systems‐level mapping of these networks, advancing understanding of coordinated RNA regulation and its disruption in disease.

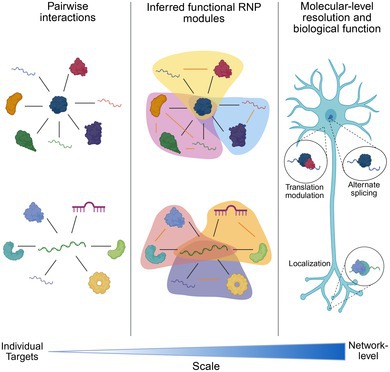

**Abbreviations**
PPI: protein–protein interaction
PTRN: post‐transcriptional regulatory network
RBP: RNA‐binding protein
RIP: RNA‐interacting protein
RNP: ribonucleoprotein

## Introduction

**Fig. 1:**
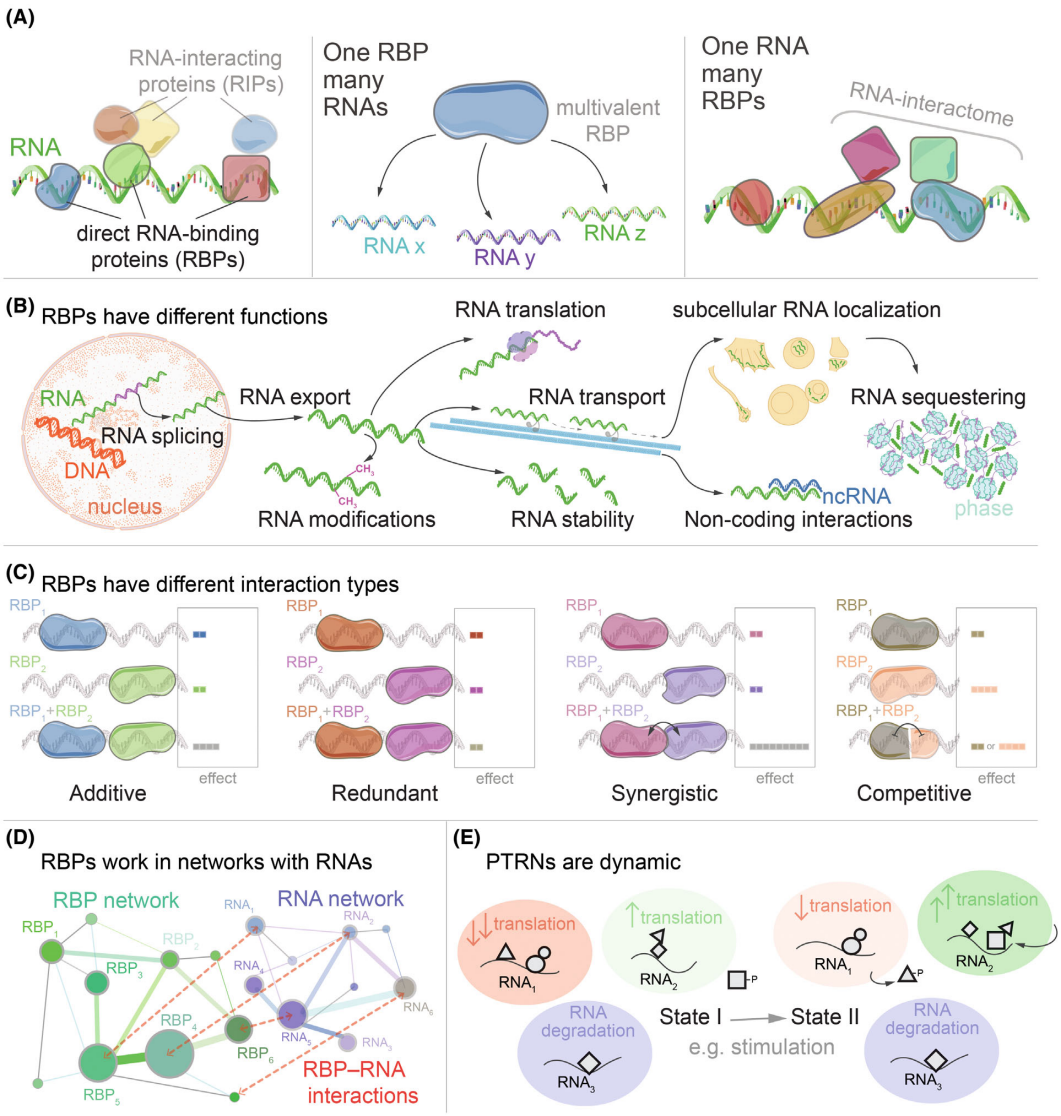
Multifaceted nature of ribonucleoprotein (RNP) composition, function, and regulation. (A) Left: The term ‘RBP’ generally refers to proteins that directly bind RNA. In a broader functional sense, it can also include RBP‐interacting proteins (RIPs), which influence RNA fate without directly binding RNA. Middle: RBPs are multivalent and can bind multiple RNA targets. Right: In addition, each RNA interacts with many RBPs. (B) RBPs have a wide range of functions throughout the RNA life cycle. (C) RBPs can interact with different outcomes. (D) RNAs and RBPs form dynamic interconnected physical and functional bipartite networks. (E) PTRNs are dynamic and change in composition, modifications, and functional outcomes across states.

RNA‐binding proteins (RBPs) mediate post‐transcriptional gene expression regulation across all stages of an RNA's life including splicing, export, localization, translation, and decay (Fig. 1) [1, 2, 3, 4, 5]. Significant efforts to catalog RBPs—through RNA pulldowns, screens, and computational approaches—have identified or predicted between 1500 and 6000 RBPs encoded in the human genome [6, 7, 8, 9, 10, 11]. While RBPs are traditionally defined as proteins that make direct contact with RNA, for many of the proposed RBPs this remains untested. In this review, we therefore use a broader definition that also encompasses RBP‐interacting proteins (RIPs), since these act within the same ribonucleoprotein (RNPs) complexes and can exert important regulatory effects on RNA metabolism (see Fig. 1A). Further complicating the study of RBPs is the large diversity of RNA molecules (mRNA, rRNA, tRNA, lncRNA, etc.); while we largely focus on mRNA here, many approaches discussed have been used to investigate other types of RNA, and understanding how these other RNA types function in RNPs is vital to creating a full picture of gene expression regulation.

As our understanding of the regulatory functions, RNA‐binding profiles, and protein–protein interactions (PPIs) of RBPs has grown, it has become clear that RBPs rarely act alone. It is not just that one RBP has the capacity to bind to many different RNA transcripts, but also one RNA molecule is often bound by multiple RBPs that work cooperatively or in competition to regulate the transcript's fate [12, 13, 14, 15, 16, 17] (Fig. 1A). For example, for a subset of mRNAs with AU‐rich elements in their 3′UTRs, RBPs TTP and KSRP, which negatively control mRNA stability, compete for binding sites with the RBP HuR, which stabilizes mRNAs, thereby modulating transcript stability and translation [18]. Conversely, other RBPs act synergistically; for example, Zipcode‐binding protein 1 binds the 3′UTR of β‐actin to localize the mRNA to the cell periphery, where it is anchored to actin filaments by elongation factor 1α and locally translated [19, 20, 21, 22, 23].

These examples illustrate how an RNA's fate is governed by the combined actions of multiple RBPs, demonstrating that in order to understand RNA regulation, we need to understand RBP‐mediated combinatorial control across multiple levels of complexity. It is necessary to understand the individual, cooperative, and competitive roles of RBPs across RNA targets and binding sites, at the levels of both single RNAs and groups of RNAs (Fig. 1C). Furthermore, these regulatory mechanisms can be specific to one RNA or to sets of RNAs. For example, mRNAs encoding functionally related proteins have been shown to be coordinately regulated by the same sets of RBPs, forming RNA operons that are important for processes such as stress responses, oxidative metabolism, and disease [12, 13, 14, 15, 16].

These intricate combinatorial possibilities give rise to complex post‐transcriptional regulatory networks (PTRNs) where the RNA fate is determined by dynamic, multifaceted regulatory networks consisting of protein–protein, protein–RNA, and RNA–RNA interactions that are context‐dependent (Fig. 1D,E) [16]. Importantly, these network dynamics can manifest spatially (different subcellular compartments), temporally (across developmental or perturbation‐induced timescales), and in a condition‐dependent manner (varying according to cellular signals or environmental cues).

The context‐dependency is, experimentally speaking, a major complicating factor to understanding PTRNs. Not only do the identities of RNAs and RBPs change between conditions, subcellular localizations, and across time, but the functions of those RBPs and RNAs can also vary [15]. For example, under normal conditions, hnRNPA1 is involved in splicing and mRNA export; however, under stress conditions, it is phosphorylated and it translocates from the nucleus to the cytoplasm where it promotes cap‐independent translation of stress‐response mRNAs [24]. Such context‐specific function exemplifies the complex nature of RNA regulation, where an RBP's role can switch depending on time, state, and place.

Considering RBPs as components of dynamic networks, rather than isolated factors, is key to understanding the true functional meaning of post‐transcriptional gene expression regulation. This review focuses on current approaches being used to map RBP regulatory networks, describes emerging technologies to interrogate these networks at scale, and presents an integrative outlook on dynamic, systems‐level models of post‐transcriptional regulation.

## Current experimental approaches to map RBP interactions and networks

**Fig. 2:**
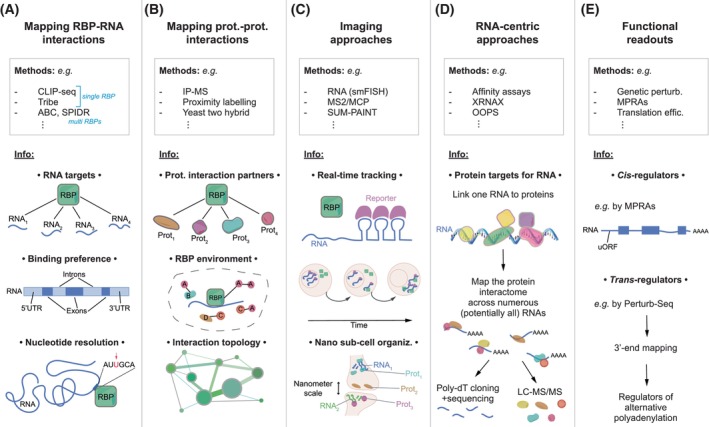
General approaches currently used to investigate PRTNs. Types of information gained from (A) mapping RBP–RNA interactions, (B) mapping protein–protein interactions of RBPs, (C) imaging approaches applied to RBPs or RNAs, (D) RNA‐centric approaches, and (E) functional readouts of RBPs or RNAs.

Elucidating RBP networks requires methods to identify which RNAs are bound by RBPs, with which other proteins RBPs interact, when and where these interactions occur in cells, and linking these interactions to defined molecular/cellular functions. A broad range of technologies has been developed for this purpose. Here, we organize them into five general categories: (a) mapping direct RBP–RNA interactions; (b) mapping the PPIs of RBPs; (c) advanced imaging approaches to visualize RNP dynamics; (d) RNA‐centric methods that, for example, pulldown RNAs and define their protein partners; and (e) high‐throughput functional screens (Fig. 2). Together, these tools are facilitating a transition from ‘anecdotal single‐factor studies’ to the comprehensive mapping of RBP network connectivity.

## The essential pair: protein–RNA interaction mapping (CLIP‐Seq and beyond)

Genome‐wide identification of the RNA targets and specific binding sites of RBPs has been foundational to RBP biology. The most widely used methods are based on crosslinking immunoprecipitation followed by sequencing (CLIP‐seq) [25, 26]. In CLIP‐seq, UV‐crosslinking in living cells covalently binds RBPs to their bound RNAs, an RBP of interest is immunopurified, and the crosslinked RNA fragments are sequenced to identify the binding sites of the RBP on its RNA targets. Variants such as HITS‐CLIP, PAR‐CLIP, iCLIP, and eCLIP, among others, have optimized different steps [27, 28, 29, 30, 31, 32, 33, 34, 35], but all have provided rich maps of RBP binding sites, often at single‐nucleotide resolution [29, 32, 36]. Systematic application of CLIP‐seq to known or predicted RBPs [17, 37], such as those performed by the ENCODE consortium, has revealed that most RBPs bind a wide array of targets—often hundreds to thousands of RNAs [37]. Furthermore, as CLIP‐seq provides binding site information, these data can also reveal the sequence motifs or RNA secondary structures that recruit the RBPs.

A more recent antibody‐based method, ARTR‐seq [38], enables the identification of RBP–RNA‐binding sites through antibody‐guided *in situ* reverse transcription of formaldehyde‐fixed cells, eliminating the need for UV crosslinking and immunoprecipitation as used in traditional CLIP‐seq. This approach allows for efficient detection of both stable and transient interactions, even in low‐input samples or tissue sections, with temporal resolutions on the scale of minutes.

*In vitro* approaches to determine the binding specificity of known or predicted RNA‐binding domains or full RBPs—such as SELEX [39, 40], SEQRS [41], RNAcompete [42], RNAcompete‐S [43], and RNA Bind‐n‐Seq [44, 45]—share a common strategy of incubating purified proteins (or protein domains) with synthetic RNA pools to identify enriched binding sequences [46]. SELEX is a classical, iterative selection method coupled with cloning and sequencing to identify high‐affinity binding motifs, but it is limited in throughput [39, 40]. SEQRS builds on SELEX by applying high‐throughput sequencing after each round of selection, significantly increasing resolution and efficiency [41]. RNAcompete is a one‐step, high‐throughput binding assay that quantifies relative binding preferences by using a vast excess of RNA over protein to increase competition across a predefined pool of approximately 250 000 sequences [42]. RNAcompete‐S [43] and RNA Bind‐n‐Seq [44, 45] expand on this by using more random and structurally diverse RNA libraries, enabling the assessment of both sequence and RNA secondary structure contributions to binding specificity.

Methods like TRIBE (Targets of RNA‐binding proteins Identified By Editing) and its derivatives offer powerful alternative *in vivo* approaches to identify RNA targets of RBPs through RNA modification [47]. In TRIBE, an RBP is fused to the catalytic domain of the ADAR enzyme, which edits adenosines to inosines near binding sites, allowing detection of target transcripts by RNA sequencing. Enhanced versions such as HyperTRIBE increase editing efficiency [48], while complementary approaches like STAMP and DART‐seq use the APOBEC1 enzyme to introduce C‐to‐U edits, providing an orthogonal readout [49, 50]. Moreover, STAMP has been shown to be able to determine the RNA targets of RBPs of interest at single‐cell resolution [49]. TRIBE‐STAMP combines both editing systems in the same cell for dual signature validation [51]. REMORA further expands this toolkit using engineered ADAR enzymes for improved specificity and programmability [52]. These techniques avoid the need for crosslinking and immunoprecipitation, enabling the study of RBP–RNA interactions in more native or low‐input contexts, though they often trade resolution for cellular compatibility and simplicity [53].

While these methods have been invaluable in advancing our understanding, they are limited to profiling one RBP at a time, meaning that significant effort is required to profile even a subset of RBPs in one condition, as the protocols are still quite labor‐intensive and also need rather large sample input amounts. Given the cooperative nature of RBP action, there is a need for approaches that can capture multiple RBPs simultaneously. Recently, high‐throughput innovations have emerged. Notably, SPIDR (split‐and‐pool identification of RBP targets) was developed as a highly multiplexed CLIP‐seq method to map the RNA targets of dozens to hundreds of RBPs in a single experiment. SPIDR uses antibody‐bead labeling coupled with split‐and‐pool barcoding to profile many RBPs at once, increasing throughput by orders of magnitude [54]. Another notable approach is ABC (antibody‐barcode eCLIP), which utilizes DNA‐barcoded antibodies and proximity ligation of the DNA oligonucleotides to RBP‐protected RNA fragments, enabling simultaneous profiling of multiple RBPs [55]. These multiplexed approaches not only accelerate data generation but also allow direct comparison of RBP binding within the same sample.

Beyond multiplexed CLIP methods, other recent techniques, such as ePRINT [56] – which uses exonuclease digestion and bioinformatics to map RBP binding sites across RBPs genome‐wide, but without ‘single RBP‐resolution’—and analysis approaches, such as Skipper [57], CLIP Tool Kit (CTK) [58] and mCross [59], have improved the statistical framework for binding site identification, thereby increasing the resolution and accuracy of RBP binding site mapping.

An exciting new avenue, and one of the ultimate goals in the field, is the precise mapping of multiple RBPs binding simultaneously to the same RNA molecule—a goal that current methods have only begun to approach. A recent example moving toward this objective is demonstrated by the combining of methods such as irCLIP‐RNP and Re‐CLIP [60], which reveals dynamic patterns of protein assemblies on RNA and co‐binding of RBPs to the same RNA molecules. As these methods continue to evolve, the field is moving toward comprehensive atlases of *when and where* on the transcript each RBP binds RNAs across the transcriptome.

## It's not all about nucleic acids: protein–protein interaction mapping

Most RBPs function within multi‐protein complexes (*e.g*., spliceosomes and translation initiation complexes) and can be members of multiple complexes or of complexes that change composition between cellular states [5, 14, 15, 61, 62]. Therefore, mapping interactions between proteins is critical to understanding RBP networks and RNA regulation. A classic interactome capture approach is immunopurification coupled to tandem mass spectrometry (IP‐MS), where an RBP of interest is immunopurified—either by the use of antibodies against the RBP of interest or against a tag in case the RBP of interest was first tagged—from cell extracts, and any co‐purifying proteins are identified by quantitative MS. This approach has revealed stable canonical complexes and a wide array of transient or condition‐specific complexes, emphasizing the need for systematic mapping of dynamic interactomes [63, 64, 65, 66, 67]. Most large‐scale interactomes do not specifically target RBPs; however, a study mapping cell‐wide interactomes and localizations found that RBPs form distinct subnetworks (based on interactions and localizations) enriched for proteins with RNA‐binding domains (RBDs) and low complexity domains [62], emphasizing the need for targeted investigations.

Two recent papers have specifically mapped the PPIs of RBPs by IP‐MS, identifying direct protein–protein and RNA‐mediated interactions across the mRNA life cycle. One in yeast [68], identified novel RBPs and secondary functions of known RBPs. The other in human cells [61] comprehensively mapped RBP interaction networks across the mRNA life cycle, identifying core RNA processing complexes, clusters of interactions within life cycle stages, and highlighted proteins bridging multiple stages or with multiple functions across the life cycle. While these studies have begun to catalog the RBP interactome, IP‐MS approaches are laborious and thus currently have been mostly limited to static conditions. In addition, IP‐MS relies on either antibodies raised against the endogenous protein, which can be expensive and difficult to find, or on tagged proteins, which are often used in overexpression constructs; furthermore, endogenous protein antibodies can sometimes interfere with protein interactions while tags and overexpression may contribute other sources of noise. In the future, advancements that enable the scaling up of interactome capture will be necessary to start mapping interactomes across different conditions or dynamic processes.

A complementary strategy to capture protein interactions is proximity labeling. In methods such as BioID and APEX, enzymes (a mutated biotin ligase or peroxidase, respectively) fused to a bait protein covalently tag proteins in the bait's vicinity within living cells, and the tagged proteins are then isolated and identified by tandem mass spectrometry (LC–MS/MS) [69, 70, 71, 72, 73]. Proximity labeling has enabled mapping of RBP–protein interactions *in situ* within specific cellular compartments or conditions. For example, the Gingras group used BioID proximity labeling to systematically map protein interaction networks across subcellular compartments and membrane‐less organelles. In one study, they generated a comprehensive subcellular proximity map using 192 markers to localize over 4000 proteins in human cells [74], and in another, they profiled 119 RNA‐associated proteins to define the composition and spatial relationships of RNA granules and processing bodies [75]. In addition, APEX‐based mapping was used to map the proteins of stress granules (both expected RBPs and novel factors) that assemble into these RNP granules during stress [76, 77, 78]. In another study, APEX was combined with phase separation to selectively enrich crosslinked RBP–RNA complexes, allowing identification of compartment‐specific RBP interactomes in living cells [79]. In a further advancement, methods such as Split‐BioID [80], Split‐TurboID [81], and sAPEX [82] split the labeling enzyme so that it is only activated upon the interaction of two proteins, thus labeling the molecular environment of a specific PPI. These proximity‐based methods capture the spatiotemporal context of RBP interactions, revealing how an RBP's protein partners can vary by location or stimulus. Advances such as shorter labeling durations and increased mass spectrometry sensitivity will increase spatial and temporal resolution, generating progressively refined maps of RBP complexes. This will potentially enhance our ability to distinguish RBPs that truly co‐assemble on the same RNA molecule from those that simply share the same cellular compartment or sequentially bind the RNA, enabling a more precise and dynamic view of RBP networks.

## Some people need to ‘*see it to believe it*’ (not us): imaging approaches

As transcript binding site identification and protein–protein interactomes are generating increasingly refined maps of RNPs, it is becoming clear that RBP‐mediated RNA regulation is complex, dynamic, and coordinated by a wide range of inputs. Thus, methods are needed that allow us to watch individual RBP interactions in cells in real time. Advanced microscopy has become indispensable for understanding the dynamic behavior of RNPs. One powerful set of techniques involves single‐molecule RNA imaging in live cells. Commonly used systems include the MS2/MCP tag, where MS2 stem‐loop aptamers inserted into an RNA of interest are bound by MCP fluorescent coat proteins [83]. Newer CRISPR‐based RNA labeling methods can track endogenous RNA molecules without tagging the RNA molecules of interest [84, 85, 86]. For example, a recent method called smLiveFISH [86], which uses the CRISPR–Csm complex to track endogenous RNAs without tagging, has been shown to decrease hands‐on time and also reduce artifacts that can arise from exogenous tagging, thereby enabling more generalizable live cell single‐molecule RNA tagging.

To complement single‐molecule RNA imaging, techniques are required to visualize RNAs and RBPs at single‐molecule resolution. The most promising methods currently used to visualize RBP–RNA interactions include smFISH, which can detect specific RBPs and RNAs in fixed cells with molecular resolution [87, 88, 89]. This method can be expanded to identify whether an RBP is bound to a target transcript by determining if the two are colocalized at the single‐RNA level [90]. Importantly, recent advancements in super‐resolution imaging such as SUM‐PAINT enable parallel labeling of up to 30 proteins and will expand our ability to visualize complex RBP assemblies *in situ* [91]. Future developments in these technologies will be especially powerful if the local structure of RBP–RNA assemblies is maintained during fixation, and if identification of mRNA species is combined with high‐resolution mapping of proteins and mRNA, allowing for simultaneous analysis. Taken together, these methods allow researchers to visually track individual mRNA molecules and potentially their bound proteins as they move and localize within the cell, providing a direct window into the spatiotemporal logic of RBP–RNA regulation and transforming abstract interaction maps into dynamic, observable phenomena. Crucially, imaging also reveals how these interactions differ across subcellular compartments, linking differences in local molecular regulation to spatially defined subcellular functions.

## Let's take the RNA's point of view: RNA‐centric technologies

Most of the large‐scale mapping approaches discussed above are based around profiling the interactions of RBPs of interest. In contrast, RNA‐centric approaches identify proteins bound to RNAs of interest. Many of the RBP cataloging studies used techniques such as poly(dT) pulldowns, OOPS, and XRNAX—in which RNA pulldown or RNP isolation is followed by mass spectrometry—to identify RBPs within the proteome [7, 8, 9, 10, 11]. While poly(dT) pulldowns specifically target the protein interactome mainly of polyadenylated RNAs [11], methods like OOPS [8] and XRNAX [7] exploit the biochemical property that protein–RNA crosslinked complexes partition to the interphase during acid guanidinium thiocyanate‐phenol‐chloroform (AGPC) extraction, enabling unbiased isolation of the global RNA‐bound proteome.

An interesting global approach to define the RNA–protein interactome over the RNA life cycle was recently performed by the Kim group, where pulse‐chase labeling of RNA molecules—via SLAM‐seq—over time was combined with quantitative proteomics to provide a time‐resolved map of RBP binding to transcripts across mRNA life cycle stages in an unbiased manner, even providing for some RNP complexes temporal resolution of their assembly [92].

However, RNA‐centric techniques can also be used in more targeted ways to understand RBP cooperation on the same RNA species and discover RBPs associated with a specific RNA of interest (e.g., a key regulatory mRNA or a long noncoding RNA). One common strategy is RNA antisense purification (RAP–MS) [93], ChIRP–MS [94], and related methods, in which biotinylated antisense oligonucleotides are used to fish out a target RNA species from crosslinked cell lysates. The pulled‐down RNA–protein complexes are then analyzed by LC–MS/MS to identify the cohort of RBPs on the target RNA. This approach has been successfully applied to numerous RNAs. For instance, RAP–MS maps of the Xist long noncoding RNA revealed many proteins that assemble on Xist to mediate X‐chromosome inactivation [93]. Similarly, RNA pulldowns targeting SARS‐CoV‐2 viral RNA identified over a hundred human proteins that specifically bind the viral RNA, including both well‐known and unexpected RBPs [95]. Another recent RNA‐centric method, Oligonucleotide‐mediated proximity‐interactome mapping (O‐MAP), uses FISH‐like probes to deliver proximity‐biotinylating enzymes to target RNAs *in situ*, enabling precise identification of nearby biomolecules (e.g., proteins and RNA molecules) without genetic manipulation [96]. As such, O‐MAP provides a multi‐omic view of RNA‐associated proteins, transcripts, and genomic loci.

It is important to note that these techniques use ensemble measurements. This implies that the identification of two RBPs on the same RNA species does not necessarily indicate co‐binding to the same molecules. However, in the future, clever experimental designs that complement these techniques with single‐complex resolution approaches will address these limitations.

## Let's get some idea of what is actually happening: high‐throughput functional screens and assays

As RNP interactome maps are becoming more comprehensive, researchers are interested in the functional consequences of these interactions. To address functional questions more comprehensively, systematic functional screens are being used more widely. High‐throughput genetic screens (CRISPR or RNAi) can pinpoint RBPs that affect a particular readout (*e.g*., a fluorescent reporter for mRNA splicing or stability, or some cellular features of interest such as stress granules). For example, the Yeo group used a pooled CRISPR screening approach combined with microraft array imaging (CRaft‐ID) to identify RBPs that regulate stress granule formation, demonstrating how image‐based genetic screens can reveal previously uncharacterized RBP functions beyond traditional proliferation or survival assays [97]. Combining single‐cell transcriptomics (scRNA‐seq) with genetic perturbations, and RBP perturbation specifically, can profile how knocking down or editing an RBP affects gene expression at high throughput, which helps to infer RBP function, while potentially even accounting for cell‐to‐cell variability [15, 98, 99]. For example, the Satija group developed CPA‐Perturb‐seq [99]. In this targeted single‐cell CRISPR screen, they perturbed 42 known and potential cleavage and polyadenylation (CPA) factors, which revealed how distinct components of the nuclear RNA processing machinery regulate polyA site selection. Other recent screens have used more elaborate reporter constructs, for example to identify splicing factors and determine if their binding has positional effects on various alternative splicing events [100].

The assays described above assess the function of trans‐acting factors, namely RBPs. However, complementary approaches exist to investigate the cis‐regulatory elements—specific RNA sequences that serve as platforms for regulation. Among these, massively parallel reporter assays (MPRAs) offer a powerful method to systematically test the regulatory activity of RNA cis elements. In MPRAs, tens of thousands of synthetic RNA sequences (e.g., 3′UTR variants) are introduced into cells, and their effects on gene expression—for example, via transcript abundance or translation efficiency—are measured *en masse*. By analyzing which sequences drive high or low expression, one can infer both the regulatory sequence elements and the likely RBPs involved [101, 102, 103, 104, 105, 106]. For instance, an MPRA might reveal that inserting multiple copies of a certain motif leads to transcript destabilization, pointing to an RBP that recognizes that motif as the likely causal factor. Coupling MPRA results with RBP motif catalogs allows potential assignment of candidate RBPs to each regulatory effect [102]. Complementary to MPRA assays, the Zhang group recently introduced SpliceRUSH, a high‐throughput CRISPR–dCas13d/gRNA screening approach that maps splicing‐regulatory elements by competitively binding to RNA regions, revealing how these elements control, for example, exon inclusion in their native sequence context [107].

In sum, high‐throughput functional screening technologies provide a powerful and complementary perspective to the four mechanistic pillars discussed above. While the first four pillars emphasize the molecular interactions that underlie post‐transcriptional regulation, high‐throughput functional assays begin to reveal the biological consequences of these interactions at scale. Each of the five pillars contributes essential and distinct insights, but none alone is currently sufficient to fully capture the complexity and context‐dependence of post‐transcriptional gene regulation.

## The biochemist might not like it, but cell lines are *in vitro*: *in vitro* versus *in vivo*

In RBP biology, early discovery and method development are very often achieved in simplified cellular systems, where experimental control is easier to achieve and scaling up is simpler. However, moving from cells to tissues and whole organisms *in vivo* is considerably more challenging. Simplified *in vitro* conditions might bias physiological stoichiometry, mask cellular heterogeneity, and limit spatial, developmental, or activity‐dependent regulation. As a result, interactions that may appear robust *in vitro* may be actually rewired, buffered, or entirely absent in native tissues, particularly in complex systems such as the nervous system. A broad range of technologies has been developed to elucidate RBP networks at the cellular level, and while most of the above‐mentioned methods were initially applied in cell lines, many have also been adapted for use *in vivo* in animal models or in human or mammalian tissues. For example, methods that identify RBP binding sites on target RNAs (CLIP‐seq, TRIBE, etc.) have been used across a range of tissues and animal models [35, 38, 47, 108]. Methods that conversely identify RBPs bound to specific sequences of interest (SELEX, O‐MAP, etc.), as well as general RBP identification methods that catalog RNA‐interacting proteins (XRNAX, SLAM‐seq, etc.), have also been modified or applied for *in vivo* applications [11, 96, 109, 110, 111, 112, 113]. Protein–protein interaction capture methods (IP‐MS, proximity labeling, etc.) and proteomics methods to capture post‐translational modifications (PTMs) or proteome turnover have been well established in tissue samples [114, 115, 116, 117, 118, 119, 120, 121, 122, 123, 124]. Finally, imaging approaches, including single molecule (smFISH) and live imaging (MS2/MCP tagging) have been demonstrated in model organisms such as C. elegans and zebrafish, with smFISH additionally being applied to tissues [125, 126, 127], while large‐scale screens (CRISPR, RNAi) have been used across model organisms, including mammals [128, 129]. Continuing methodological advances will increase our ability to more completely map RBP networks *in vivo*, mirroring the information that can currently be captured for *in vitro* cellular systems.

## Outlook: toward integrative and dynamic models of RBP networks

As the toolkit for studying RBPs has grown, so too has our appreciation of the dynamic, systems‐level nature of post‐transcriptional gene expression regulation. We have now reached a turning point where descriptive, static datasets can give way to integrative and predictive models of RBP networks. Several challenges define the road ahead.

## From snapshots to movies: spatiotemporal regulation and conditional specificity

**Fig. 3:**
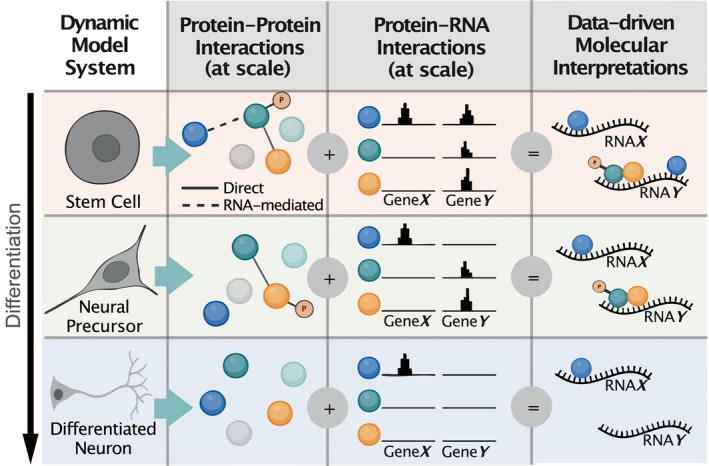
Data integration across time, datasets and molecular layers. To picture the dynamics of RBPs during a biological process (*e.g*., neuronal differentiation), complementary datasets that capture distinct aspects need to be combined. For example, integration of two different molecular layers (protein–RNA and protein–protein interactions) enables data‐driven interpretations of how specific RBPs, post‐translational modifications, and protein–RNA contacts cooperate to regulate distinct transcripts at each stage of the biological process of interest.

Most existing data about RNP networks provide a snapshot of RBP interactions under one condition. The next step is to capture how these interactions change over time and/or between cellular states. This will, for example, require the integration of methodologies that track RBP–RNA and RBP–protein interactions across different stimuli or developmental stages and eventually in live cells (Fig. 3).

For example, time‐resolved CLIP or pulse‐chase labeling could monitor the dynamics of RBP binding during a cellular transition such as stress response or cell differentiation. These types of data might be achieved via some form of combined RNA labeling and CLIP, such as CLIP on samples with short pulses of 4‐thiouridine (4sU) RNA labeling followed by different chase times, or CLIP against target RBPs that have been tagged with an inducible proximity‐labeling enzyme [80, 82, 130] where only those RNAs bound during the proximity‐labeling pulse are labeled. From the proteome perspective, time‐resolved proteome profiling can be used to capture the stepwise assembly and disassembly of RBP complexes, as demonstrated by a recent study of stress granule formation and dissolution [76]; one important requirement for such approaches, however, will be the ability to synchronize the cellular process being investigated as the data will still reflect ensemble measurements. By incorporating the temporal dimension, we move from static networks to ‘movies’ of RBP interactions, revealing transient players and timing cues that are invisible in endpoint assays.

To achieve ‘movie’‐like RNP resolution spatial information is needed in addition to temporal information. RBPs function in specific subcellular locales—the composition of an RNP in the nucleus can differ from that on the same RNA in the cytoplasm—and RBPs can have multiple spatially‐distinct functions [62]. For example, in addition to its canonical splicing role in the nucleus, the splicing factor SNRNP200 has a secondary stress granule‐associated role in the cytoplasm, with distinct protein interactors, binding motifs, and RNA targets [61]. Spatially resolved RBP networks can be generated using techniques such as compartment‐specific interactome capture [79] or *in situ* RNA proximity labeling [77]. Integrating these data across cellular compartments will enable mapping distinct subnetworks of RBPs operating in the nucleus, cytosol, stress granules, neuronal axons, etc., with each context having its own set of RBP interactions and regulatory logic.

An important factor that will need to be incorporated into studies using both currently available methods and future approaches is the function of RNA modifications and PTMs of RBPs. There are countless individual examples of how these modifications alter the function, localization, and interaction partners of RNAs and RBPs in response to a wide range of stimuli [131, 132]. Consequently, these RNA and protein modifications add an additional dimension of complexity to the project of achieving ‘movie’‐like resolution of PTRNs that needs to be addressed. Modifications can be taken into account via the use of antibodies against modified protein residues or RNA nucleotides or by direct methods to measure them. This information can be mapped directly—for example, to identify the PPIs or RNA targets of a modified protein, or to identify the RNA modifications within a subcellular compartment—or layered with other methods such as imaging approaches that follow modified residues in relation to other molecules in real‐time and at single‐molecule resolution. RNA modifications can be measured at single‐nucleotide resolution by chemical reactivity methods, such as MAZTER‐seq [133], m6A‐SAC‐seq [134] and GLORI [135] for m6A; Pseudo‐seq [136, 137], Ψ‐seq [138] and PSI‐seq [139] for pseudouridine; and bisulfite sequencing for m5C [140]. However, these methods rely on the accessibility of the modified residue so RBP‐bound modifications, for example, would not be captured if these methods were combined directly with current standard CLIP‐seq approaches. Inventive work‐arounds will need to be developed to be able to generate such multi‐layered data. Similarly, PTMs can also be measured at the proteome‐wide scale by enrichment (*e.g*., phospho‐enrichment) followed by MS, and these measurements can, for at least some modification sites, identify which specific residues are modified. While recent progress has increased our ability to map PTMs onto protein complexes and their effect on PPIs [141], we still lack the suite of tools that will enable comprehensive mapping of PTMs and identification of their impact on molecular interactions across dynamic processes. For example, tagging a PTM‐recognition domain, or RNA modification reader protein with a proximity‐labeling enzyme would enable spatial and temporal labeling of the neighborhood around modifications and would be a useful tool that could be combined with other methods to determine the PPIs and RNA interactions of RBPs. Looking forward, such novel methods will need to be developed to enable the capture of modifications in their molecular context within RNPs and across time, space, and at high resolution.

The ultimate goal will be to gather a four‐dimensional atlas (3D space over time) of RNP interactions in cells. Achieving this will require both experimental innovation (to label and observe RNAs and proteins at high spatiotemporal resolution) and computational tools to integrate multi‐dimensional data and, potentially, fill temporal gaps that cannot be solved experimentally. As an example, one could imagine an integrated model of an mRNA's journey: from being co‐transcriptionally bound by splicing RBPs in the nucleus, handed off to export factors, then to cytoplasmic stabilizers or translators, and eventually to decay machinery—all orchestrated by a network of RBPs that engage at the right place and time. These atlases can then be complemented by additional information about the functional consequences of these interactions, such as the turnover rates of RNAs and proteins [123, 124, 142, 143, 144, 145], which will add an additional quantitative measure of the temporal dynamics—one which is becoming increasingly relevant in disease and aging contexts. Developing and expanding ‘4D atlases’ across cell types, stimuli responses, development, and disease states seems a big challenge, but it will be required to understand how cells rewire post‐transcriptional regulation to meet physiological demands. Yet, with the accelerating pace of technological and computational advances, this ambitious vision is gradually becoming an achievable reality.

## Integration and computational modeling

As the volume of data from large‐scale omics approaches continues to grow, a major challenge is the integration of multi‐omics datasets into coherent network models that can reveal the functional logic of post‐transcriptional regulation. Such integrative analyses have, for example, clarified the splicing networks controlled by the Nova and Rbfox families [146, 147], and provided insights into how alternative splicing is regulated during neuronal development and across distinct neuronal cell types [148, 149]. In addition, a recent multi‐lab study demonstrated the power of this multi‐omics strategy by defining functional regulatory modules of RBPs through the integration of protein interaction data, RBP–RNA‐binding maps, and transcriptomic changes following RBP perturbations [15]. This integrative framework enabled the inference of causal relationships—identifying groups of RBPs that jointly regulate specific transcripts or pathways, and showing how the composition and activity of these RBP modules can shift in a context‐specific manner, with individual RBPs participating in distinct regulatory complexes depending on cellular conditions. This work serves as a blueprint for future multi‐omics approaches aimed at deciphering RBP function [15].

Recent experimental advances have made it feasible—even for individual laboratories—to generate matched datasets that include RBP–RNA and RBP–protein interactions, along with transcriptomic responses to RBP perturbation, setting the stage for the integrative analyses discussed above. Machine learning is already playing—and will continue to play—a central role in extracting insight from these complex, multi‐dimensional datasets. Foundational AI approaches like AlphaFold [150] have dramatically improved our ability to predict protein structure and, increasingly, PPIs. While accurate prediction of protein–RNA interaction and even PPI for RBPs remains challenging, the rapid growth in available training data is expected to significantly improve model performance in the coming years—potentially enabling prediction of dynamic interaction changes across different cellular states. Early success in this direction has already been demonstrated [151, 152]. For example, deep neural networks such as HDRNet can integrate sequence, structure, and experimental binding data to predict RBP binding profiles and how they vary across cell types and conditions [152]. In parallel, expanding on existing graph‐based approaches that have been used to model RBP–RNA interactions as bipartite networks [15, 17, 37], will enable the identification of key regulatory nodes, co‐regulated RNA modules, and patterns of combinatorial control. As more high‐resolution datasets become available and computational models grow more sophisticated, these approaches will be essential for distilling complexity into testable hypotheses.

Additionally, despite rapid technological progress, it remains a challenge to reconcile insights obtained from *in vitro* or simplified cellular systems with the behavior of RBP networks *in vivo*. In addition to future experimental advancements, integrative and computational modeling frameworks provide a natural avenue to address this gap by explicitly treating RBP interactions as context‐dependent and probabilistic rather than fixed. By integrating data across experimental systems, perturbation regimes, and biological scales, such models can identify which interactions are conserved across contexts, which are condition‐specific, and how network states shift between *in vitro* and *in vivo* environments. Ultimately, closing this gap will require not only more physiologically relevant datasets but also models that can accommodate abstraction, uncertainty, and emergent behavior when extrapolating from controlled experimental systems to intact organisms.

Importantly, all these integrated network analyses are indeed revealing that many RBPs are pleiotropic, performing multiple, context‐dependent roles rather than acting through a single, fixed mechanism. Future models will need to account for this functional modularity and complexity, embracing the dynamic and conditional nature of RBP activity in diverse cellular environments.

## Higher throughput and completeness

An important ongoing trend is the increase in throughput and breadth of RBP studies. To truly capture systems‐level networks, we will eventually need to cover most (if not all) RBPs and their interactions. Projects like ENCODE have mapped >350 RBPs by CLIP, but hundreds more await profiling [37]. New high‐throughput techniques (such as multiplexed CLIP via SPIDR [54] or ABC [55]) will accelerate the completion of the RBP–RNA interactome, potentially allowing routine profiling of many RBPs in a cell type within one experiment. The future development of the split‐and‐pool approach used in SPIDR, for example, could potentially address the problem of capturing interactions at single‐complex resolution at a large scale. This could be achieved by increasing the dimensionality of the captured complexes from an RBP and its RNA targets to multiple RBPs binding the same RNA target by more completely labeling the components of the RNPs.

Similarly, systematic RBP–protein interactomes (using approaches like high‐throughput IP‐MS [153] or proximity labeling of many baits in parallel) will fill in the missing edges in the network. As these interaction datasets become more comprehensive and capture diverse cell states and cell types, they will feed into better integrative models and also highlight the most physiologically significant interactions. Notably, with greater throughput comes the ability to examine perturbations in combinations: rather than only knocking down one RBP at a time, researchers can begin to perturb pairs or sets of RBPs to systematically test how network nodes function together. This will help move beyond correlation to causation in RBP networks.

## Toward predictive understanding

A long‐term goal of RNP biology is to develop a predictive, quantitative understanding of post‐transcriptional regulatory networks on par with transcriptional networks. In transcription, we now have predictive models for how combinations of transcription factors control gene expression programs [154, 155, 156]. For RBPs, achieving similar predictive power would mean we could, given a set of RBPs and an RNA, forecast the stability, localization, or translation of that RNA in a given context. This will likely require both data‐driven and mechanistic modeling. On one hand, large integrated datasets analyzed using statistical or mathematical frameworks—or with the help of AI‐driven approaches—may uncover recurring patterns or network motifs (such as feed‐forward loops, feedback loops, and competitive modules) in RBP networks, pointing to underlying general principles of post‐transcriptional regulation. On the contrary, detailed kinetic modeling of particular RNP assemblies (in the spirit of systems biology models for metabolic or signaling networks) could quantify how an mRNA's half‐life or translation rate emerges from the binding and action of multiple RBPs.

Encouragingly, we are already seeing cases where multi‐RBP models improve understanding—for instance, models of ARE‐mediated mRNA decay that include both stabilizing and destabilizing RBPs can explain ARE‐mediated gene expression changes in processes such as inflammation better than single‐factor models [18], and mRNA levels can be better modeled by taking into account the co‐regulatory cross‐talk of RBPs in transcription and decay [157]. As more experimental feedback refines these models, we move closer to a true systems biology of RNA. In such a framework, one could simulate how a cell's RBP network state changes under stress or disease, and what post‐transcriptional effects would ensue.

## Disease

Embracing the complexity of RBP networks is not just an academic exercise to try to understand gene expression regulation; it has practical implications for human health. Many RBPs are being investigated as therapeutic targets in diseases, such as in cancer and neurological disorders. However, a recurring challenge is that targeting one RBP can have unforeseen, pleiotropic effects or limited efficacy, owing to the redundant and compensatory nature of RBP networks [158]. For instance, in cancer, an RBP that stabilizes pro‐oncogenic transcripts might seem like an ideal drug target, but if another RBP can bind the same transcripts when the first is inhibited, the cancer cell may bypass the drug's effect. This partly explains why some drugs or antisense oligonucleotides against single RBPs have shown modest success: the interconnected network dampens the impact.

Similarly, in neurodegenerative diseases like ALS or FTD, pathogenesis involves RNP granule dysregulation with multiple RBPs (TDP‐43, FUS, etc.) misbehaving; a one‐gene‐one‐phenotype therapeutic approach often falls short in such multifactorial scenarios [159, 160]. Thus, the complexity of RBP regulatory networks and potential off‐target effects are major challenges in developing RBP‐targeted therapies [158, 161]. Going forward, strategies that account for the network—for example, targeting a critical node that affects multiple RBPs, or modulating a downstream process common to a network of RBPs—may be more fruitful. Alternatively, combinatorial interventions (akin to multi‐drug regimens) might be needed to simultaneously tweak several RBPs or pathways.

## Conclusions

In conclusion, the study of RBPs is evolving from cataloging individual protein–RNA and PPIs to deciphering an integrated network of RNP regulation. Evidence now shows that RBPs operate in a highly interconnected, dynamic manner with spatiotemporal and conditional differences. By leveraging emerging technologies—from multiplexed binding assays and high‐resolution imaging to single‐cell and computational integration multi‐omics datasets—our community begins to be equipped to map and study these RNP networks with unprecedented depth and clarity.

Future steps are ambitious: rather than static lists of interactions, we aim to assemble 4D dynamic functional models that explain how RBPs cooperate and compete over time to fine‐tune gene expression in changing cellular environments. Such models will not only enrich fundamental understanding of post‐transcriptional gene regulation, but may also offer insights into disease mechanisms and treatments and suggest new ways to modulate gene expression by targeting RBP interactions. In summary, RBPs should be seen not as solo actors but rather as nodes in dynamic, context‐responsive networks that we can now begin to comprehensively chart.

## Conflict of interest

MJ is an inventor on a submitted patent application related to SPIDR (US application no.: 18/665401), a method discussed in this article.

## Author contributions

LS, MJ, and EFF wrote, edited, and revised the manuscript.
